# Organellar DNA Polymerases in Complex Plastid-Bearing Algae

**DOI:** 10.3390/biom9040140

**Published:** 2019-04-07

**Authors:** Yoshihisa Hirakawa, Arisa Watanabe

**Affiliations:** 1 Faculty of Life and Environmental Sciences, University of Tsukuba, 1-1-1 Tennodai, Tsukuba, Ibaraki 305-8572, Japan; 2 Graduate School of Life and Environmental Sciences, University of Tsukuba, 1-1-1 Tennodai, Tsukuba, Ibaraki 305-8572, Japan; arisa321w@gmail.com

**Keywords:** algae, chlorarachniophytes, DNA replication, endosymbiosis, mitochondria, plastids

## Abstract

DNA replication in plastids and mitochondria is generally regulated by nucleus-encoded proteins. In plants and red algae, a nucleus-encoded enzyme called POP (plant and protist organellar DNA polymerase) is involved in DNA replication in both organelles by virtue of its dual localization. POPs are family A DNA polymerases, which include bacterial DNA polymerase I (PolI). POP homologs have been found in a wide range of eukaryotes, including plants, algae, and non-photosynthetic protists. However, the phylogeny and subcellular localizations of POPs remain unclear in many algae, especially in secondary and tertiary plastid-bearing groups. In this study, we report that chlorarachniophytes possess two evolutionarily distinct POPs, and fluorescent protein-tagging experiments demonstrate that they are targeted to the secondary plastids and mitochondria, respectively. The timing of DNA replication is different between the two organelles in the chlorarachniophyte *Bigelowiella natans*, and this seems to be correlated to the transcription of respective POP genes. Dinoflagellates also carry two distinct POP genes, possibly for their plastids and mitochondria, whereas haptophytes and ochrophytes have only one. Therefore, unlike plants, some algal groups are likely to have evolved multiple DNA polymerases for various organelles. This study provides a new insight into the evolution of organellar DNA replication in complex plastid-bearing organisms.

## 1. Introduction

Plastids and mitochondria originated from independent endosymbiotic events, wherein cyanobacteria-like and α-proteobacteria-like organisms became fully integrated with their host eukaryotes [1]. Although mitochondria were derived from a single endosymbiotic event, plastids evolved through multiple endosymbioses. A common ancestor of Archaeplastida, which includes plants, green algae, red algae, and glaucophytes, obtained a primitive plastid through the uptake of a cyanobacterial endosymbiont [2,3]. Subsequently, green and red algae were enslaved by phagotrophic protists in several different lineages [4,5]. These events are called “secondary endosymbioses”, and have led to the evolution of diverse organisms with secondary plastids in the eukaryotic tree of life (i.e., haptophytes, ochrophytes, cryptophytes, chromerids, dinoflagellates, apicomplexans, euglenophytes, and chlorarachniophytes). Furthermore, there were “tertiary endosymbioses”, wherein some phagotrophic dinoflagellates discarded their original plastids and gained new endosymbionts by engulfment of haptophytes and diatoms [6]. Although the exact number of endosymbiotic events is still under debate, multiple independent and serial endosymbioses have led to the diversity of modern-day plastid-bearing organisms [7].

Plastids and mitochondria maintain their own genomes, which have descended from the original bacterial endosymbionts; however, these organellar genomes lack most genes for DNA replication (e.g., DNA polymerases, DNA primase, and DNA helicases), except for the plastid-encoded DnaB helicase in some algal lineages [8,9]. Thus, organellar genome replication is catalyzed by nucleus-encoded proteins. In plants, plastid and mitochondrial DNA are synthesized by a nucleus-encoded DNA polymerase. They have sequence homology to bacterial DNA polymerase I (PolI) and belong to the family A of DNA polymerases [10,11,12,13]. Such PolI-like sequences have been found in diverse eukaryotes, including plants, algae, and non-photosynthetic protists [14]. These organellar DNA polymerases are, therefore, called “POP” (plant and protist organellar DNA polymerase). As an exception, apicomplexan parasites and chromerids have another PolI-like protein that is distinct from POP, and is known to be involved in DNA replication of their non-photosynthetic secondary plastids (apicoplasts) [15,16,17]; this protein is referred to as “PREX” (plastidic DNA replication/repair enzyme complex). In contrast, opisthokonts (animals and fungi) use DNA polymerase γ (Polγ) for mitochondrial DNA replication, and Polγ shows low similarity to bacterial PolI [18,19]. In terms of structure, POP sequences typically contain a 3′-5′ exonuclease and DNA polymerase domain, differing from bacterial PolI in its lack of a 5′-3′ exonuclease domain [20]. PREXs carry a primase and helicase domain in their N-termini, in addition to a 3′-5′ exonuclease and DNA polymerase domains [16].

According to previous phylogenetic studies, POP sequences form a monophyletic clade that is not closely related to the PolI sequences of cyanobacteria and α-proteobacteria [20]. Given the conservation of POP across diverse eukaryotic lineages, POP probably originated in an ancestral eukaryote. It is worth noting that the replication of bacterial chromosomes is primarily performed by the DNA polymerase III (PolIII) holoenzyme in *Escherichia coli* [21]. This suggests that an original DNA replicase was replaced by POP when bacterial endosymbionts became organelles. In contrast, PREX sequences have a close relationship with bacterial genes encoding a bifunctional 3′-5′ exonuclease and DNA polymerase, which differs from PolI [17]. PREXs were, therefore, probably derived from a certain bacterial lineage by lateral gene transfer. In addition to POP and PREX, DNA polymerase θ (Polθ) and DNA polymerase ν (Polν) are also family A DNA polymerases, and have a function in mammalian nuclear DNA repair [22,23].

It has been reported that nucleus-encoded POPs are dually targeted to plastids and mitochondria in several photosynthetic organisms. In the land plants *Arabidopsis thaliana* and *Nicotiana tabacum*, two closely related copies of POP genes have been found, and their protein products are targeted to both plastids and mitochondria [10,12]. The red alga *Cyanidioschyzon merolae* has a POP gene, the products of which are also localized in both organelles [24]. Moriyama et al. (2008) reported that secondary plastid-bearing diatoms, *Phaeodactylum tricornutum* and *Thalassiosira pseudonana*, also possess a single gene for POP [20], inferring its dual localization to the secondary plastids and mitochondria. However, localization experiments of POP proteins are limited in a few model organisms, and organelle DNA polymerases remain undetermined in many secondary plastid-bearing algae.

In this study, we searched POP homologs using available genome and transcriptome data, and identified them in diverse algal groups. In addition to diatoms, five secondary algal groups, chlorarachniophytes, cryptophytes, chromerids, dinoflagellates, and haptophytes, were found to carry POP genes. Interestingly, chlorarachniophytes possess two phylogenetically distinct POPs that are targeted to the plastids and mitochondria, respectively. Phylogenetic analyses suggested that the mitochondria-targeted POP is of host origin, and the plastid-targeted POP was probably derived from an ancestral lineage related to ochrophytes through lateral gene transfer. Furthermore, the timing of DNA replication appeared to be different between the plastids and mitochondria in chlorarachniophytes, which may be related to the transcription of their respective POP genes. In other secondary plastid-bearing algae, haptophytes had a single POP gene, possibly suggesting that their POPs are dually targeted into both organelles. However, dinoflagellates possessed two distinct copies of POP, similar to chlorarachniophytes. These data suggest that organellar DNA replication may be regulated by a dual-localized POP in plants and diverse algae. Meanwhile, some secondary plastid-bearing algae, such as chlorarachniophytes, have evolved two distinct POPs that are individually targeted into plastids and mitochondria.

## 2. Materials and Methods 

### 2.1. BLAST Search of POP Homologous Genes

Genome data covering major lineages of eukaryotes, Metazoa, Fungi, Viridiplantae, Choanozoa, Amoebozoa, Excavata, Heterokonta, Haptophyta, Cryptophyta, Rhizaria (including Chlorarachniophyta), Apusomonadida, and Alveolata were obtained from the GenBank database (https://www.ncbi.nlm.nih.gov/), the Joint Genome Institute (JGI) Genome Portal site (https://genome.jgi.doe.gov/portal/) and EnsembleProtists (http://protists.ensembl.org/index.html). A total of 678 transcriptomes by the Marine Microbial Eukaryotic Transcriptome Sequencing Project (MMETSP) were downloaded from iMicrobe (https://www.imicrobe.us/) [25]. For the chlorarachniophyte *Bigelowiella natans*, we applied refined gene models generated by a previous study [26]. Potential homologous sequences of POPs and PREXs were surveyed in these datasets by BLAST using the POP sequence of *Arabidopsis thaliana* (At1g50840) and the PREX sequence of *Plasmodium falciparum* (XP_001348285) as a query, with a cut-off E-value ≤ 1 × 10^−5^.

### 2.2. Phylogenetic Analysis

Our collected sequences were combined with the sequences of family A DNA polymerases used in previous phylogenetic studies [17,20]. All sequences were subjected to a preliminary phylogenetic analysis. After removal of redundant and excessively partial sequences, a total of 131 sequences (93 POPs, 7 PREXs, and the other family A DNA polymerases) were used for rigorous phylogenetic analyses. The sequences were automatically aligned by the L-INS-I method of the MAFFT package [27], and gaps and poorly-aligned positions were trimmed by trimAl with the automated1 option [28], resulting in 463 amino acid positions. Maximum likelihood phylogenetic analyses were performed with IQ-TREE [29] under an empirical mixture model (LG+C20+F+Γ) [30], as well as the best-fitting model (LG+R7) that was selected by ModelFinder implemented in IQ-TREE [31]. To use the LG+C20+F+Γ model, the guide tree was obtained by the simpler LG+F+Γ model. Branch support values were evaluated with 100 standard non-parametric bootstrap replicates.

### 2.3. Subcellular Localization of Chlorarachniophyte POPs

To investigate the subcellular localization of chlorarachniophyte proteins, we used green/yellow/cyan fluorescent protein (GFP/YFP/CFP) tagging. Total RNA was extracted from the chlorarachniophyte *B. natans* (CCMP621) using TRIzol reagent (Invitrogen, Carlsbad, CA), and cDNA was synthesized using ReverTra Ace (Toyobo, Osaka, Japan) with random primers. The cDNA fragments encoding an N-terminal presequence of BnPOP1 (the first 112 of 809 amino acids, accession number: LC463047) and BnPOP2 (the first 351 of 1251 amino acids, accession number: LC463046) were amplified by PCR, with specific primers adding a restriction site. Each fragment was inserted into pLaRGfp+mc [32] or pLaRYfp+mc vector using *Hin*dIII and *Nco*I. As a mitochondrial marker, we generated the vector expressing a mitochondrial histidine tRNA synthetase fused to CFP by modifying the HisRS77124-2ndMet+GFP vector [33]. These plasmids were cloned in the DH5α strain of *Escherichia coli* and purified with a QIAprep Spin miniprep kit (Qiagen, Hilden, Germany). As there are no available transformation systems for B. natans, we transformed the related chlorarachniophyte species, *Amorphochlora amoebiformis*, with these plasmids using a Biolistic PDS-1000/He particle delivery system (Bio-Rad), as described previously [34]. Twenty-four to 48 h after bombardment, transformed cells were observed under an inverted Zeiss LSM 510 laser scanning microscope (Carl Zeiss, Jena, Germany).

### 2.4. In Silico Prediction of Targeting Signals and Functional Domains

After removal of partial sequences lacking an N-terminal methionine, 49 POP/PREX sequences were subjected to in silico prediction. N-terminal targeting signals (i.e., mitochondrial targeting peptide, chloroplast transit peptide, and signal peptide) were predicted using TargetP 1.1 [35], Predotar 1.04 [36], and PredSL [37]. Hydrophobic signal peptides were also predicted using TMHMM 2.0 [38] as a potential transmembrane helix in their first 60 amino acids. Functional domains of POPs were searched using the HMMER server (http://hmmer.org/) with the Pfam HMM database.

### 2.5. Real-time Quantitative PCR

Cell division of *B. natans* was temporally synchronized under white light conditions in 12:12 light:dark cycles, as previously described [39]. The cell number was monitored using a hemocytometer. Cells were collected by centrifugation from 100 mL cultures every 4 h during a diurnal cycle (at 0, 4, 8, 12, 16, 20, and 24 h). DNA and RNA extractions were performed using a Plant DNA Preparation Kit (Jena Bioscience, Jena, Germany) and TRIzol Reagent (Invitrogen), respectively; we failed to purify DNA from the sample at 16 h.

To estimate the amount of DNA for each genome at each time point, we performed quantitative PCR (qPCR) using specific primer sets (BnN#1 and BnN#2 for the nuclear genome; BnM#1 and BnM#2 for the mitochondrial genome; BnP#1 and BnP#2 for the plastid genome) designed in a previous study [40]. Real-time qPCR was carried out using a Thermal Cycler Dice Real-Time System II (Takara, Kusatsu, Japan). Reactions contained 1 µL DNA solution, 0.4 µM of each primer, and 12.5 µL of SYBR Premix Ex Taq II (Takara), and the volume was made up to 25 µL with DNase/RNase-free water. The cycling conditions included 3 min of denaturation at 95 °C, followed by 40 cycles of 10 s at 95 °C, and 30 s at 60 °C. A melting curve program was used for the detection of nonspecific products. Target fragments were cloned into the pGEM-T easy vector (Promega, Madison, WI), and standard curves were generated using serially diluted solutions of plasmids. The relative copy number of the target DNA was calculated using Ct values (2nd derivative maximum).

To estimate the transcript levels for BnPOP1 and BnPOP2, cDNA was synthesized from total RNA using a ReverTra Ace qPCR RT Kit (Toyobo). Real-time qPCR was performed under the same conditions as described above, using specific primer sets (BnPOP1-F: GTAGTCGTAGCAGGGCATCTTGT, BnPOP1-F: CCCTCTCCTCCTCACATCAATC, BnPOP2-F: AGTAGGAGCAACAACGCAGTAGG, BnPOP2-R: GGCTGGAGTTGGATAGGATTTG, Bn18S-F: TGCCAGGCGATAGTTCATTC, Bn18S-R: TTGGATGTGGTAGCCGTTTC) designed using the Primer3Plus online software. Relative transcript levels of BnPOP1 and BnPOP2 at each time point were calculated by the ΔΔCT method [41], using 18S rRNA as an internal control. Real-time qPCR was repeated three times to estimate standard deviations.

## 3. Results

### 3.1. Identification of POP Genes in Diverse Algae and Protists

**Figure 1:**
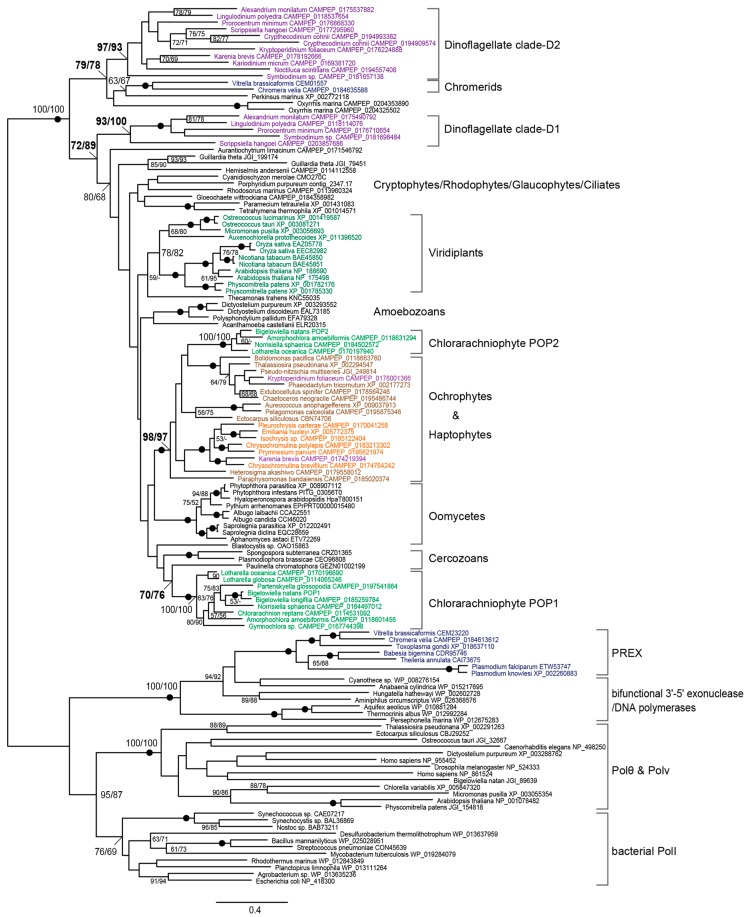
Maximum likelihood phylogenetic tree of family A DNA polymerases, including 93 POPs (plant and protist organellar DNA polymerases). The tree was generated under the LG+R7 model implemented in IQ-TREE. The values at nodes indicate bootstrap supports calculated by the two different substitution models (LG+R7/LG+C20+F+Γ) when they are higher than 50%, and black dots on branches represent robust supports (>95%). The scale bars show the number of inferred amino acid substitutions per site. Accession numbers of GenBank, JGI (Joint Genome Institute)/, and MMETSP (Marine Microbial Eukaryotic Transcriptome Sequencing Project) are shown on the right side of species names.

Based on homology searches in available genomic and transcriptomic databases, we identified homologous genes for the organellar DNA polymerase POP in diverse eukaryotes. Based on Pfam searches, all POP sequences analyzed here were predicted to consist of a 3′-5′ exonuclease and DNA polymerase domain. A single gene of POP existed in both primary and secondary plastid-bearing algae (e.g., chlorophytes, rhodophytes, glaucophytes, haptophytes, and ochrophytes), as well as non-photosynthetic protists, including ciliates, oomycetes, amoebozoans, and rhizarians. However, no genes for POP were detected in opisthokonts and kinetoplastids. Land plants, as well as the cryptophyte *Guillardia theta*, non-photosynthetic protists, *Oxyrrhis marina*, and *Crypthecodinium cohnii*, possessed two closely-related copies of POP, probably reflecting a recent gene duplication in each species or lineage (Figure 1). On the other hand, two distantly-related POP genes were found to exist in chlorarachniophytes and photosynthetic dinoflagellates with secondary and tertiary plastids.

Chlorarachniophytes have acquired plastids through a secondary endosymbiosis between a cercozoan protist and a green alga. POP genes were searched in chlorarachniophytes using the genome data of *B. natans* and transcriptome data from 8 species. We found that 4 species carried two distinct POP genes, whereas the others had one. We constructed phylogenetic trees using two substitution models, LG+R7 and LG+C20+F+Γ, and both of which showed similar tree topologies. Based on our phylogenetic tree, 9 POPs (referred to as POP1) found in all species form a monophyletic clade with 100% bootstrap support (BS). This takes a sister position to the cercozoan *Paulinella chromatophora* with 70%/76% BS of the LG+R7/LG+C20+F+Γ model (Figure 1). By contrast, the other 4 POPs (POP2) are closely related to ochrophyte POPs, and the clade consisting of ochrophyte, haptophyte, and chlorarachniophyte POPs is robustly supported with 98%/97% BS (Figure 1). The two clades of chlorarachniophyte POP1 and POP2 are distantly related to each other, and neither showed a close affiliation with green algae.

Two POP genes were also found in dinoflagellates with peridinin-containing plastids that were derived by secondary endosymbiosis of red algae. These POPs appear in separate clades, named “clade-D1” and “clade-D2”, which are supported with 93%/100% and 97%/93% BP, respectively. Both dinoflagellate clades are located at the basal position of all POPs (Figure 1). Clade-D1 is occupied by peridinin-containing dinoflagellates, whereas clade-D2 includes non-photosynthetic species (Figure 1). Two distinct POPs were also found in tertiary plastid-bearing dinoflagellates, *Karenia brevis* and *Kryptoperidinium foliaceum*, whose plastids were derived from a haptophyte and a diatom, respectively. One of their POPs was included in clade-D2, and the others branched within the haptophyte and diatom clade, respectively (Figure 1). In contrast to peridinin-containing dinoflagellates, POPs of two non-photosynthetic species (*Noctiluca* and *Crypthecodinium*) that likely lack plastid genomes were found only in clade-D2 (Figure 1).

Apicomplexan parasites possess PREX for apicoplast DNA replication instead of POP. Interestingly, the photosynthetic relatives of apicomplexans, *Vitrella brassicaformis* and *Chromera velia*, carry genes for both PREX and POP. The phylogenetic tree indicates that their POPs are most closely related to that of the dinoflagellate relative *Perkinsus marinus*, albeit with weak supports (63%/67% BP) (Figure 1).

### 3.2. Localization Experiments for Chlorarachniophyte POP Proteins

**Figure 2:**
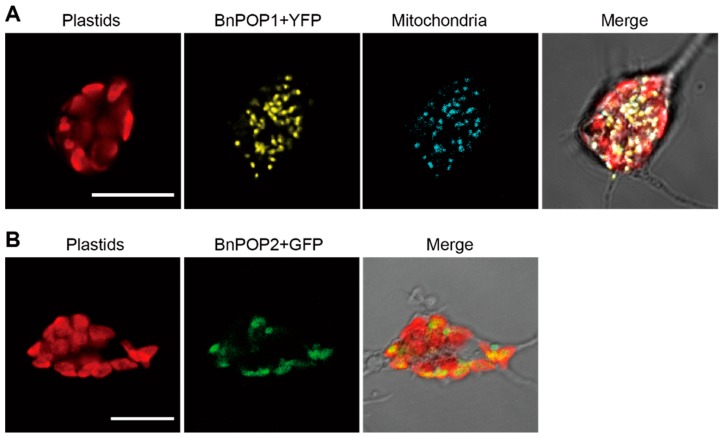
Subcellular localization of BnPOP1 and BnPOP2. Green/yellow/cyan fluorescent protein (GFP/YFP/CFP) fusion proteins were expressed in the chlorarachniophyte, *Amorphochlora amoebiformis*. The red color is the chlorophyll-autofluorescence, and the yellow and green signal represent localization of BnPOP1 (**A**) and BnPOP2 (**B**), respectively. Mitochondria were labelled by CFP fused with the mitochondria-targeted histidine tRNA synthetase. The scale bars are 10 µm.

We performed fluorescent protein tagging to reveal the subcellular localization of two evolutionarily distinct POPs in the chlorarachniophyte *B. natans*. Based on in silico analyses, the *B. natans* POP1 (BnPOP1) was predicted to have a mitochondrial targeting peptide by TargetP and PredSL, whereas BnPOP2 was found to carry an N-terminal hydrophobic domain, similar to a signal peptide of typical secondary plastid-targeted proteins. When the YFP fusion protein with the N-terminal sequence of BnPOP1 was heterologously expressed in the chlorarachniophyte *Amorphochlora amoebiformis*, yellow fluorescence was observed in many small dots in the cytoplasm. These dots were co-localized with the mitochondrial maker (HisRS77124-2ndMet+CFP) (Figure 2A). On the other hand, GFP with the N-terminal sequence of BnPOP2 was co-localized with chlorophyll autofluorescence (Figure 2B). Therefore, nucleus-encoded BnPOP1 and BnPOP2 appeared to be targeted to mitochondria and plastids, respectively.

### 3.3. Timing of Organellar DNA Replication in B. natans

**Figure 3:**
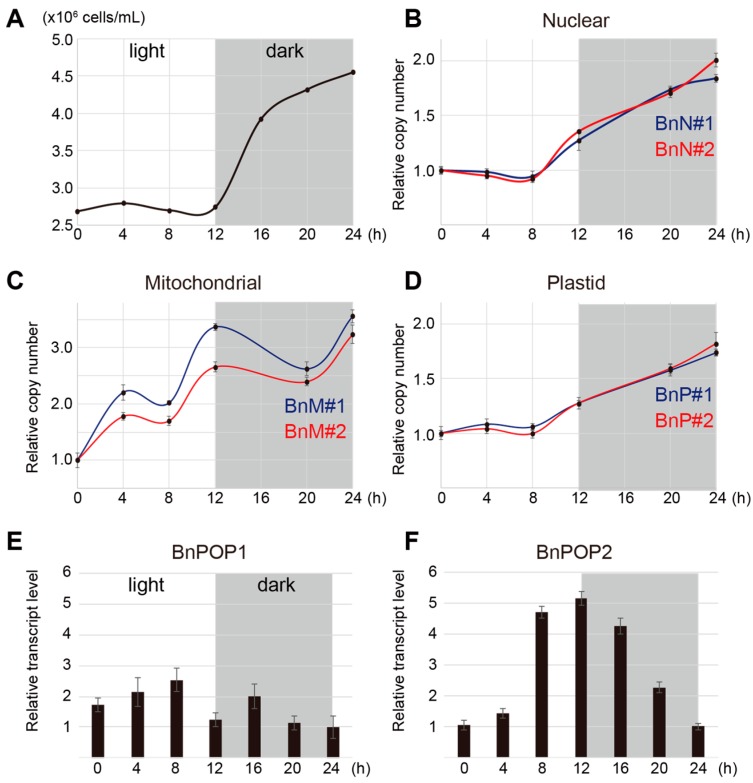
Timings of DNA replication for nuclear, mitochondrial, and plastid genomes. (**A**) Cell growth in temporally synchronized culture of *Bigelowiella natans* during a diurnal cycle. (**B**–**D**) Plots of changes in nuclear, mitochondrial, and plastid DNA levels, which were determined by real-time qPCR with two sets of specific primers (blue and red). (**E**,**F**) Relative transcript levels for BnPOP1 and BnPOP2. Error bars represent the standard deviation of triplicate qPCR reactions.

To estimate the timing of organellar DNA replication in a diurnal cycle, we extracted DNA from temporally synchronized *B. natans* cells at 4 h intervals, and performed real-time qPCR using specific primer sets for the nuclear, plastid, and mitochondrial genome. In the synchronized cultures, cell division occurred only in the dark phase (Figure 3A). According to qPCR analysis, nuclear DNA levels increased during the dark phase, and its replication seemed to begin in advance of cell division (Figure 3B). The plastid DNA was also synthesized in the dark phase, similar to the nuclear DNA (Figure 3D). On the other hand, an increase of mitochondrial DNA was observed in both the light and dark phases, and the timing of its replication was ambiguous (Figure 3C). The data on the timing of DNA replication were inferred from the single cycle, due to the temporary synchronization of *B. natans* cells. We also performed qPCR to calculate the transcript levels of BnPOP1 and BnPOP2 at each time point. BnPOP2 transcripts reached a peak level at dusk and represented the lowest level at dawn (Figure 3F). However, BnPOP1 transcript levels were not drastically changed in the diurnal cycle, and the transcript levels in the light phase were slightly higher compared to the dark phase (Figure 3E). Taken together, the transcription pattern of BnPOP2 seems to be consistent with the timing of plastid DNA replication, and the constant levels of BnPOP1 transcripts did not conflict with the fact that mitochondrial DNA are synthesized through the diurnal cycle.

### 3.4. Prediction of Targeting Signals in POP Sequences

We performed in silico prediction of N-terminal targeting signals in 43 POPs after removal of partial sequences (supplementary Table S1). In chlorarachniophytes and their relatives, POPs of the chlorarachniophyte *Gymnochlora* sp. and the cercozoan protist *Plasmodiophora brassicae* were predicted to have a mitochondrial targeting peptide by the three prediction servers (TargetP, Predotar, and PredSL). These sequences are phylogenetically close to the mitochondria-targeted BnPOP1 (Figure 1). In haptophytes, the POP of *Emiliania huxleyi* was predicted to have a signal peptide, and an aromatic amino acid (tryptophan) was found at the +1 position of the potential cleavage site, similar to typical targeting signals for secondary plastids of red lineages. However, if translation is initiated at the 2nd possible methionine (at the +19 position), the product was predicted to be targeted to mitochondria. Similarly, the POP of another haptophyte *Isochrysis* sp. was predicted to carry a signal peptide from the first methionine and a mitochondrial targeting peptide from the second methionine at the +9 position. In contrast to haptophytes, any typical targeting signals were not estimated in ochrophyte POPs. In non-photosynthetic species of ciliates, oomycetes, and amoebozoans, most sequences were predicted to carry a mitochondrial targeting peptide. Unfortunately, many dinoflagellate POPs were not incorporated into signal prediction, due to the absence of their N-terminal ends. In the tertiary plastid-bearing dinoflagellate *Karenia brevis*, the POP sequence in clade-D2 was predicted to be targeted to mitochondria, and the other one in the haptophyte clade was found to have a putative signal peptide, similar to plastid-targeted proteins. Additionally, a couple of sequences in the dinoflagellate clade-D2 were predicted to possess a mitochondrial targeting peptide. However, any sequences of clade-D1 could not be applied, due to the absence of N-terminal ends.

## 4. Discussion

### 4.1. Evolution of Two Distinct POPs in Chlorarachniophytes

The organellar DNA polymerase POP has been reported as a dual-targeted protein to both plastids and mitochondria in land plants and a red alga [10,12,24]. In this study, we found that the chlorarachniophyte *B. natans* carried two phylogenetically distinct POPs (BnPOP1 and BnPOP2), which were targeted to mitochondria and plastids, respectively. Two types of POP were detected in the MMETSP transcriptome data of three chlorarachniophytes (*Amorphochlora amoebiformis*, *Lotharella oceanica*, and *Norrisiella sphaerica*), whereas the other five species were found to carry only a POP1 gene. This is likely because their transcriptome data do not represent all protein-coding genes, and would lack part of the periodically expressed transcripts, including POP2. Indeed, two POP genes were obtained in the JGI genome data of *B. natans*, while its transcriptome (MMETSP0045 from iMicrobe) did not represent one of them. Mitochondria-targeted POP1 sequences showed a close phylogenetic relationship with the cercozoan *P. chromatophora* (Figure 1), and the POP of another cercozoan *Plasmodiophora brassicae* carries a putative mitochondrial targeting peptide. This suggests that mitochondrial POP1 was inherited from the host through secondary endosymbiosis. In contrast, plastid-targeted POP2 sequences branch within the clade of ochrophytes and haptophytes possessing red algal secondary plastids (Figure 1). Considering the green algal origin of chlorarachniophyte plastids, POP2 may be derived from an ancestral lineage related to ochrophytes/haptophytes via lateral gene transfer.

Our findings raise the question of why two organellar DNA polymerases were evolved in chlorarachniophytes. One possible reason is that DNA replication might be separately controlled in plastids and mitochondria by transcription of distinct POP genes. The mitochondrial and plastid DNA of *B. natans* are synthesized at different times during the cell cycle, which seems to be correlated with transcript changes of BnPOP1 and BnPOP2, respectively (Figure 3). In the unicellular red alga *C. merolae*, the kinetics of DNA replication are different between plastids and mitochondria, although DNA synthesis in both organelles is likely to be catalyzed by a dual-localized POP [42]. This suggests that the transcription of POP gene is not a limiting factor for regulating the timing of organellar DNA replication in *C. merolae*. It has been reported that the activity of plastid POP is regulated by the redox state in the green alga *Chlamydomonas reinhardtii*. In vivo and in vitro experiments have indicated that plastid DNA replication was activated and inactivated by artificially changing the cellular redox state, though the POP protein level was constant [43]. In some primary plastid-bearing algae, it is therefore possible that dual-localized POPs are independently activated by the redox state in each organelle, causing kinetic differences in organellar DNA replication. Taken together, regulatory mechanisms of organellar DNA replication in chlorarachniophytes might differ from those in other photosynthetic organisms possessing dual-targeted POPs.

### 4.2. POP Evolution in Alveolates

Similar to chlorarachniophytes, dinoflagellates were found to have two distinct POPs. POPs of peridinin-containing dinoflagellates formed two clades, clade-D1 and clade-D2, both of which are located at the basal position of all POPs in the phylogenetic tree (Figure 3). Based on the tree, we could not conclude that genes of clade-D1 and clade-D2 are the result of a duplication from an ancestral POP gene. The in silico prediction of targeting sequences suggested that POPs in clade-D2 would be mitochondrial DNA polymerases. Although there is no localization information for clade-D1 POPs, we can infer that they may function as a plastid DNA polymerase. This idea does not conflict with the fact that clade-D1 consists of only photosynthetic dinoflagellates, while clade-D2 also contains plastid genome-lacking species. Secondary plastid-bearing dinoflagellates are likely to have evolved two POP genes for plastids and mitochondria, but localization experiments for their products are necessary for further elucidation.

In tertiary plastid-bearing dinoflagellates, *K. brevis* possesses two POPs branching within the dinoflagellate clade-D2 and the haptophyte clade, respectively. Interestingly, the haptophyte-related POP sequence was predicted to have a plastid-targeting signal. Through the tertiary endosymbiosis, in which *K. brevis* replaced the original peridinin-containing plastid with the engulfed secondary plastid of haptophytes [44], the original plastid-localized POP would have been replaced with the endosymbiont-derived POP. This gene replacement may be linked to changes of plastid genome architecture. Genomes of peridinin-containing plastids are composed of multiple small circular molecules, dubbed minicircles, each of which generally encode a single gene [45]. By contrast, the haptophyte-derived plastid genome is most likely a typical single circular chromosome [46], though the complete plastid genome sequence is unavailable in *K. brevis*. This structural difference is possibly linked to the replacement of plastid DNA polymerases.

We have found that the chromerids, *V. brassicaformis* and *C. velia*, have genes for both POP and PREX. Their PREX forms a monophyletic clade with apicoplast-targeted PREXs, as reported previously [17], and their POPs are most closely related to the putative mitochondria-targeted POP of the dinoflagellate relative *P. marinus*. Although dinoflagellates have two POPs for plastids and mitochondria, apicomplexan parasites have no POPs, which have been replaced by an apicoplast-targeted PREX and an uncharacterized mitochondrial polymerase, respectively. Chromerids, therefore, appear to represent an intermediate stage between apicomplexans and dinoflagellates, as their plastid-targeted POP would have been replaced by PREX, while the putative mitochondrial POP seems to be retained. However, localization experiments for the chromerid POP and PREX are necessary for further discussion.

It has been reported that the POP of the ciliate *Tetrahymena thermophila* is localized to mitochondria [14]. Our in silico prediction revealed that most POP sequences found in protists (e.g., Amoebozoa, Apusozoa, Ciliophora, Oomycota, Perkinsozoa, and Rhizaria) carry a putative mitochondrial targeting peptide (supplementary Table S1). This suggests that mitochondrial POPs exist not only in ciliates, but also in diverse lineages of non-photosynthetic protists.

### 4.3. POP Evolution in Haptophytes and Ochrophytes

Two haptophytes, *E. huxleyi* and *Isochrysis* sp., were found to have a single gene for POP. Those POP sequences were predicted to carry uncanonical N-terminal targeting signals that encode a putative plastid-targeting signal from the first methionine and a putative mitochondrial targeting peptide from the second methionine. Therefore, it is tempting to suggest that two protein isoforms from a single POP gene are generated by alternative translation, and that they are targeted to plastids and mitochondria in haptophytes, respectively. A similar dual targeting system controlled by alternative translation was reported for aminoacyl-tRNA synthetases of secondary plastid-bearing algae [33,47]. In contrast to haptophytes, all POP sequences of ochrophytes analyzed here were estimated to have no typical targeting signals. We cannot eliminate the possibility that those POP sequences are partial and lacking proper N-terminal ends, which leads to incorrect localization perdition. However, another possible interpretation is that ochrophyte POPs function in nuclei rather than plastids and mitochondria, and if so, as yet unknown organellar DNA polymerases may exist.

## 5. Conclusions

The bacterial PolI-like polymerase POP is found in diverse eukaryotes, including plants, algae, and non-photosynthetic protists. In plants and red algae, POP is involved in the DNA replication of plastids and mitochondria, and products from a single POP gene are known to be dually targeted to both organelles. In this study, we reported that chlorarachniophyte algae have evolved two phylogenetically distinct POPs that are targeted to the plastids and mitochondria, respectively. This finding indicates that photosynthetic organisms have evolved organelle DNA polymerases in two different directions: either “one polymerase for both organelles” or “one polymerase for each organelle”. However, POP localization remains unconfirmed in many organisms, and further localization experiments will be necessary to understand the whole picture of organelle DNA polymerase evolution.

## Supplementary Materials

The following are available online at https://www.mdpi.com/2218-273X/9/4/140/s1. Table S1: In silico prediction of subcellular localization of POPs.
